# Integrated multi-omics profiling identifies aging-related molecular signatures and convergent interferon signaling in systemic lupus erythematosus

**DOI:** 10.3389/fimmu.2026.1861904

**Published:** 2026-08-19

**Authors:** Hongwei Zeng, Donge Tang, Yong Dai, Qinxin Zhang, Wenhan Du, Jing Du, Haiyan Yu, Jiayu Gu, Wei Zhang

**Affiliations:** 1 Department of Clinical Laboratory, Peking University Shenzhen Hospital, Shenzhen, China; 2 Department of Experimental Research, South China Hospital, Medical School, Shenzhen University, Shenzhen, China; 3 Clinical Medical Research Center, The First Affiliated Hospital (Shenzhen People’s Hospital), Southern University of Science and Technology, Shenzhen, China; 4 The First Affiliated Hospital, School of Medicine, Anhui University of Science and Technology, Huainan, China; 5 Department of GCP, The Third People’s Hospital of Shenzhen, The Second Affiliated Hospital of Southern University of Science and Technology, Shenzhen, Guangdong, China; 6 Department of Pharmacy, Shenzhen People’s Hospital (The Second Clinical Medical College, Jinan University; The First Affiliated Hospital, Southern University of Science and Technology), Shenzhen, China

**Keywords:** aging-related pathways, interferon signaling, kinase networks, phosphoproteomics, proteomics, systemic lupus erythematosus (SLE)

## Abstract

**Background:**

Systemic lupus erythematosus (SLE) is characterized by chronic immune activation and molecular alterations that overlap with aging-related biological processes. However, how these alterations are organized across molecular layers and whether they converge on shared regulatory networks remain incompletely understood.

**Methods:**

We performed an integrative multi-omics analysis combining in-house proteomic and phosphoproteomic data from 130 patients with SLE and 90 healthy controls (HCs) and publicly available transcriptomic datasets comprising 1,461 SLE patients. Proteins and phosphorylation sites were annotated using established aging-related gene resources. Differential protein abundance and phosphorylation changes were analyzed across disease-status and disease-activity comparisons. Nominal *P*-value thresholds were used for exploratory feature selection, whereas FDR-adjusted *P* values were used to assess robustness after multiple-testing correction. Kinase-substrate enrichment, transcription factor annotation, and cell-type-resolved transcriptomic comparison were used to explore potential regulatory programs.

**Results:**

We identified 128 nominally altered proteins annotated to aging-related biological processes, including genomic instability, mitochondrial dysfunction, and epigenetic alterations. Phosphoproteomic analysis revealed 36 nominally altered phosphorylation sites, including previously unreported sites in IFI16 (S153, S780) and PKCδ (S507, S664). Clustering analysis demonstrated heterogeneous protein co-regulation patterns across disease states. Kinase activity inference suggested altered activity of TBK1 and IKKβ. TF analysis further highlighted STAT1, RELA, and PML as potential central nodes within the inferred regulatory network. Notably, these multi-omic alterations were not randomly distributed but showed convergence toward shared signaling pathways, particularly those related to interferon responses.

**Conclusions:**

This integrative multi-omics study identifies inflammatory and interferon-dominated molecular alterations in SLE PBMCs that overlap with aging-related biological processes and converge on shared regulatory networks. These findings provide a hypothesis-generating framework for investigating the intersection between chronic immune activation and aging-related molecular remodeling in SLE.

## 1 Introduction

Systemic lupus erythematosus (SLE) is a complex autoimmune disease characterized by chronic systemic and multi-organ inflammatory responses. Increasing evidence suggests that SLE shares several biological features with aging, including impaired immune-cell function, mitochondrial dysfunction, epigenetic alterations, and disrupted immune homeostasis (1, 2). These observations raise the possibility that aging-related molecular alterations contribute to SLE pathogenesis, although their overall molecular landscape remains poorly characterized.

At the molecular level, SLE-associated immune dysregulation involves several processes that also occur during aging, including aberrant activation of type I interferon and NF-κB signaling, chromatin instability, epigenetic dysregulation, mitochondrial dysfunction, and oxidative stress (3–5). Most studies focusing on individual pathways or molecular events, and it remains unclear whether these diverse alterations are organized in a coordinated manner at the systems level.

Protein phosphorylation is a central mechanism regulating intracellular signaling in immune cells. In SLE, key inflammatory pathways such as type I interferon, NF-κB, and MAPK are largely controlled by aberrant phosphorylation events (6–8). Phosphoproteomics more directly reflects dynamic signaling activity and kinase-associated regulation compared with transcriptomic or proteomic measurements, and may help infer upstream regulatory networks underlying cellular signaling processes. Integrating proteomic and phosphoproteomic profiles therefore provides complementary information for characterizing signaling alterations and regulatory network changes in SLE.

In this study, we aimed to characterize SLE-associated proteomic and phosphoproteomic alterations that overlap with aging-related biological processes and to determine whether these alterations converge on shared regulatory programs. Compared with previous SLE phosphoproteomic studies that primarily focused on phosphorylation landscapes and kinase-regulated networks (9), we integrated proteomic, phosphoproteomic, and public transcriptomic data using aging-related biological processes as an interpretative framework. We further performed disease-status-associated analyses and inferred upstream kinase and transcription factor (TF) networks to provide a systems-level view of the molecular pathways linking SLE-associated immune dysregulation with aging-related biology.

## 2 Methods

### 2.1 Transcriptomic data acquisition

Publicly available transcriptomic data were obtained from the Gene Expression Omnibus (GEO) database, including datasets GSE65391, GSE121239, GSE61635, GSE4588, GSE50772, GSE99967, and GSE24706, comprising 1,461 patients with SLE and 198 HCs. Detailed information regarding the included datasets has been reported in our previous study (10).

Single-cell RNA-sequencing data generated from our previously established SLE cohort (11) were used to provide cell-type-resolved transcriptomic context for the bulk PBMC proteomic findings. The raw and processed scRNA-seq data are publicly available in the GEO under accession number GSE158263.

Aging-related genes were curated through integration of aging-associated gene sets from Gene Set Enrichment Analysis (GSEA)-based pathway resources and the National Genomics Data Center (NGDC) Aging Atlas database (https://ngdc.cncb.ac.cn/aging/age_related_genes) (**Supplementary Table 1**).

### 2.2 Differential expression analysis of public transcriptomic data

The transcriptomic datasets were processed separately, including log2 transformation and between-array normalization. Genes shared across datasets were retained, and dataset-associated batch effects were corrected using ComBat in the R package “sva”. Differential expression between SLE and healthy controls (HCs) was subsequently assessed using the R package “limma” with empirical Bayes moderation. Genes with an absolute log2 fold change >0.585 and Benjamini-Hochberg-adjusted *P* values < 0.05 were retained for subsequent analysis.

### 2.3 Proteomic and phosphoproteomic datageneration and preprocessing

The proteomic and phosphoproteomic datasets analyzed in the present study were previously generated from an in-house PBMC cohort and reported in our earlier study (9). The source cohort included 90 HCs, 82 patients with inactive SLE, and 48 patients with active SLE. All patients with SLE fulfilled the Systemic Lupus International Collaborating Clinics (SLICC) classification criteria (12). Disease activity was assessed using the Systemic Lupus Erythematosus Disease Activity Index 2000 (SLEDAI-2K) based on both clinical and laboratory parameters (13). Patients with a SLEDAI score ≤4 were classified as inactive-stage SLE (SLE_S), whereas those with a SLEDAI score >4 were classified as active-stage SLE (SLE_A). The demographic and clinical characteristics of HCs and patients with SLE are summarized in **Supplementary Table 2**.

For mass-spectrometry analysis, PBMC proteins were combined into analytical pools, including 15 HC pools, 14 inactive-SLE pools, and 7 active-SLE pools, with each pool comprising protein obtained from approximately six participants. Pooling was used to obtain sufficient protein input for phosphoproteomic analysis and to generate group-level analytical samples. Each analytical pool contained 1 mg of total protein. Accordingly, the analytical pools, rather than individual participants, constituted the independent units used for the proteomic and phosphoproteomic comparisons.

PBMCs were isolated from EDTA-anticoagulated peripheral blood using Ficoll-Hypaque density-gradient centrifugation and stored at −80 °C until analysis. Cells were lysed by sonication in buffer containing 8 M urea and 1% protease inhibitor cocktail. Cellular debris was removed by centrifugation at 12,000 ×g for 10 min at 4 °C, and protein concentrations were determined using a bicinchoninic acid assay.

Equal amounts of protein were reduced with 5 mM dithiothreitol at 56 °C for 30 min and alkylated with 11 mM iodoacetamide for 15 min at room temperature in the dark. Samples were diluted with 100 mM triethylammonium bicarbonate to reduce the urea concentration to below 2 M. Proteins were digested with trypsin using an enzyme-to-protein ratio of 1:50 overnight, followed by a second digestion at a ratio of 1:100 for 4 h. For phosphoproteomic analysis, phosphopeptides were enriched using immobilized metal-affinity chromatography, desalted using C18 ZipTips, and analyzed by liquid chromatography-tandem mass spectrometry.

Peptides were separated using a nanoElute UHPLC system and analyzed on a timsTOF Pro mass spectrometer using a four-dimensional label-free quantification workflow and parallel accumulation-serial fragmentation acquisition. The MS/MS scan range was 100-1,700 m/z, and 10 PASEF-MS/MS scans were acquired per cycle, with a dynamic exclusion time of 30 s.

Raw mass-spectrometry data were searched using MaxQuant version 1.6.6.0 against the human SwissProt database concatenated with a reverse-decoy database. Trypsin/P was specified as the proteolytic enzyme, with up to two missed cleavages permitted. The minimum peptide length was seven amino acids. Precursor-ion mass tolerance was set to 20 ppm, and fragment-ion mass tolerance was set to 0.02 Da. Carbamidomethylation of cysteine was specified as a fixed modification, whereas protein N-terminal acetylation, methionine oxidation, and phosphorylation of serine, threonine, and tyrosine were specified as variable modifications. The false-discovery rate (FDR) for peptide and protein identification was controlled at below 1%.

For quantitative analysis, peptide intensities were converted to relative abundance values by dividing the signal intensity of each peptide in each analytical pool by the mean intensity of that peptide across pools. For phosphoproteomic data, the relative abundance of each phosphorylation site was further normalized to the relative abundance of its corresponding protein to reduce the influence of changes in total protein expression. Quantitative values were subsequently log2-transformed before statistical analysis.

### 2.4 GSEA

In this study, differentially expressed proteins were functionally enriched using GSEA gene sets to identify biological pathways that significantly differ between patients with SLE and HCs (14). During the analysis, gene sets from multiple databases were selected, including Gene Ontology Biological Process (GO-BP) gene set, Kyoto Encyclopedia of Genes and Genomes (KEGG) pathway gene set, and HALLMARK gene set. These gene sets cover age-related pathways such as DNA repair, epigenetic regulation, oxidative stress, mitochondrial function, and intercellular communication. First, differentially expressed proteins were sequenced according to log2 fold change values for GSEA enrichment analysis. GSEA analysis parameters were set to a gene set size of 10 to 500 to minimize biases from noise genes and overly large gene sets. Enrichment Score (ES) and Normalized Enrichment Score (NES) were calculated using 1,000 permutation tests. The FDR < 0.05 was used as the significance threshold. Based on the GSEA results, the pathways that are significantly upregulated or downregulated in SLE patients were screened, with special attention to age-related biological processes such as DNA repair, mitochondrial dysfunction, and epigenetic regulation.

### 2.5 Protein-protein interaction network and subnet module identification

Protein-protein interaction (PPI) network analysis was conducted using the Metascape Online platform (https://metascape.org) (15). First, the selected differentially expressed proteins are uploaded to the Metascape platform, and through the integrated STRING database, BioGrid, IntAct and other PPI databases, the known or predicted interaction information between proteins was automatically queried. For the obtained PPI network, the interaction confidence threshold was set to filter out interactions with low confidence and ensure the reliability of the network. In addition, the Metascape platform was used to identify biologically significant subnetwork modules.

### 2.6 Hub proteins

The PPI network was constructed using the STRING database (16) and the cytoHubba plug-in of Cytoscape (17) was used in the PPI network to identify the candidate hub protein by Maximal Clique Centrality (MCC) algorithm. Candidate Hub proteins show high centrality in the network and may be involved in the regulation of biological processes.

### 2.7 Cluster analysis

Mfuzz soft clustering was performed using the SRplot online platform (https://bioinformatics.com.cn/), which implements the fuzzy c-means algorithm. Standardized group-level abundances of the aging-related proteins across HC, SLE_S, and SLE_A were clustered using nine clusters. Sensitivity analyses were additionally performed using eight, ten, and eleven clusters. Because this analysis was exploratory, the resulting clusters were used only to summarize major abundance patterns associated with SLE status or disease activity. This approach was used to identify abundance patterns across the three clinical groups rather than to infer true longitudinal or temporal dynamics.

### 2.8 Kinase activity prediction

Upstream kinases were initially predicted using iGPS 1.0 (http://igps.biocuckoo.org/) based on phosphorylation sites and surrounding sequence motifs. Kinase activity was subsequently inferred using a GSEA-based method by mapping phosphorylation data to kinase-specific substrate sets to estimate relative activity changes across conditions. Nominal enrichment *P* values were adjusted for multiple testing using the Benjamini-Hochberg method, and the corresponding FDR values were reported.

### 2.9 Kinase-substrate relationship

To further define kinase-substrate relationships, the corresponding protein with differentially phosphorylated sites were submitted to PhosphoSitePlus (https://www.phosphosite.org/homeAction.action) to retrieve putative *in vivo* upstream kinases. These were then compared with the kinases predicted in this study, and overlapping candidates were considered indicative of potential kinase-substrate relationships.

### 2.10 TF regulatory network

Experimentally validated TFs for downstream genes in blood cells were retrieved from the hTFtarget database (https://guolab.wchscu.cn/hTFtarget/) (18). Changes in these TFs between SLE and HC were assessed based on protein abundance and phosphoproteomic data. TFs exhibiting alterations in protein levels or phosphorylation were considered potential regulators in SLE.

### 2.11 Statistical analysis

All statistical analyses were performed using R version 4.3.0 and GraphPad Prism version 10.0. Protein and phosphoprotein intensities were normalized and log2-transformed before statistical analysis. For the in-house proteomic and phosphoproteomic datasets, the independent analytical units were the pooled PBMC samples, comprising 15 HC pools, 14 SLE_S pools, and 7 SLE_A pools. No individual-participant-level proteomic or phosphoproteomic measurements were used for statistical testing. Comparisons were performed between SLE_S and HCs, SLE_A and HCs, and SLE_A and SLE_S using two-sample, two-sided Student’s t-tests at the analytical-pool level. Nominal *P* values were adjusted for multiple testing within each comparison using the Benjamini-Hochberg method, and both nominal *P* values and FDR-adjusted *P* values are reported in the **Supplementary Tables 3**, **4**. Nominal *P*-value thresholds were used to identify exploratory differential features for visualization and downstream hypothesis-generating analyses. Features meeting only the nominal threshold were described as nominally altered, whereas only those meeting the prespecified FDR threshold were considered statistically robust after multiple-testing correction. Correlation analyses were performed using Spearman’s rank correlation coefficient (ρ). All statistical tests were two-sided unless otherwise specified.

## 3 Results

### 3.1 Proteomic alterations in SLE overlap with multiple aging-related biological processes

**Figure 1:**
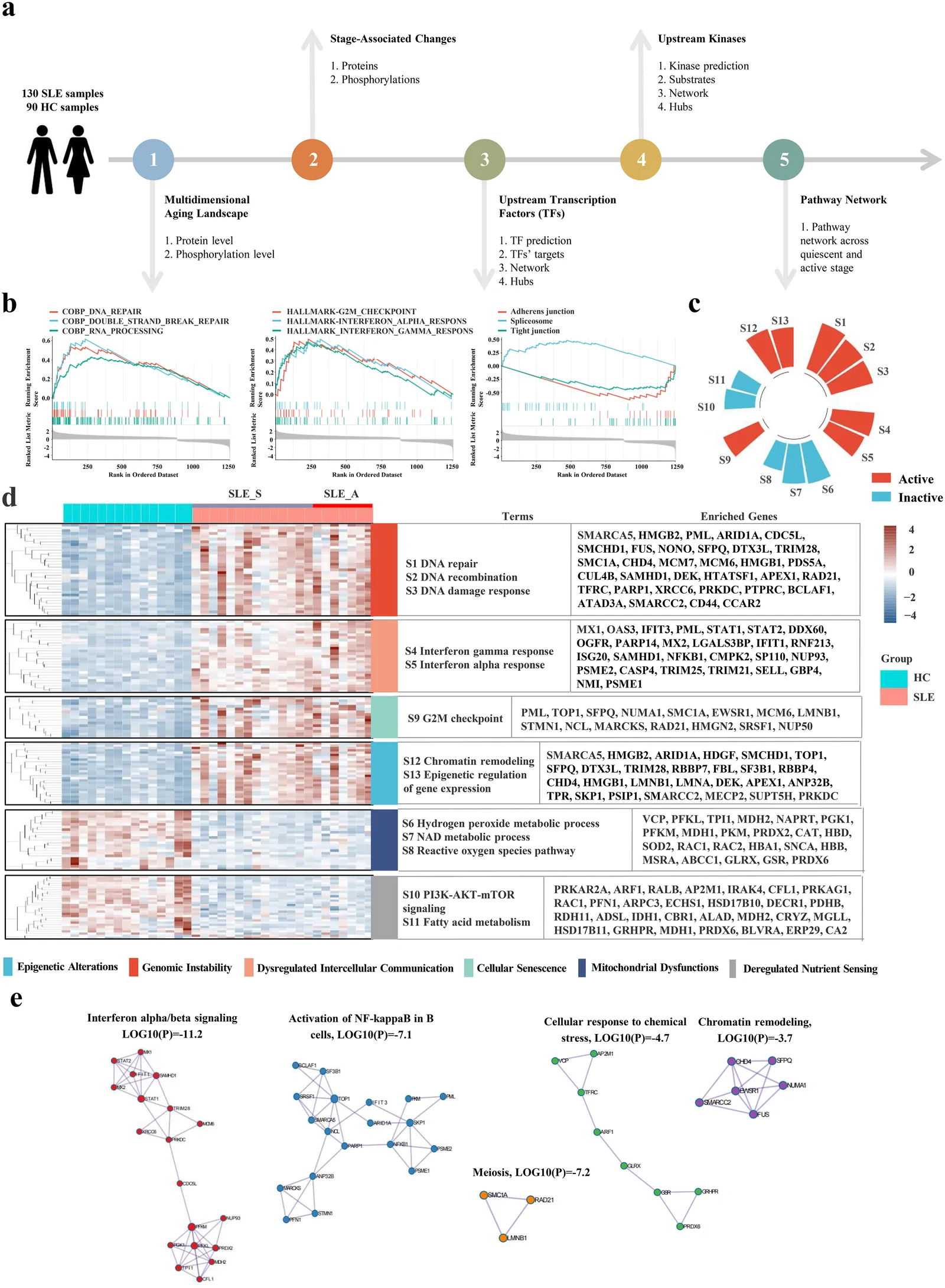
Proteomic alterations in SLE PBMCs overlapping with aging-related biological processes. **(a)** Overview of the study design and analytical workflow. The source cohort comprised 82 patients with SLE_S, 48 patients with SLE_A, and 90 HCs. For proteomic and phosphoproteomic analyses, PBMC proteins from approximately six participants within the same clinical group were combined into one analytical pool, resulting in 14 SLE_S pools, 7 SLE_A pools, and 15 HC pools. These analytical pools constituted the independent analytical units. **(b, c)** GSEA of proteomic alterations between SLE patients and HCs identified enrichment of multiple biological pathways, including DNA repair, interferon response, NAD metabolism, and antioxidant detoxification. In the GSEA plots, the x-axis represents the ranked protein list generated from the SLE versus HC comparison, whereas the y-axis represents the running enrichment score calculated by the GSEA algorithm. In panel c, red bars indicate pathways potentially activated in SLE, whereas blue bars indicate pathways potentially inactivated in SLE. Pathway-level significance was evaluated using FDR-adjusted *P* values, as described in the Methods. **(d)** Heatmap showing aging-related nominally altered proteins mapped to pathways. Differential protein abundance between SLE patients and HCs was assessed using Student’s t test, and proteins with nominal *P* < 0.05 were retained for exploratory visualization. Benjamini-Hochberg-adjusted *P* values were calculated to account for multiple testing and are provided in **Supplementary Table 3**; only proteins meeting the prespecified FDR threshold were considered robust after multiple-testing correction. Effect sizes are reported as log2 fold changes. Rows represent proteins and columns represent independent analytical pools rather than individual participants. Red and blue colors indicate relatively increased and decreased protein abundance, respectively, based on row-scaled protein abundance values. The top annotation denotes sample groups. The colored side bars indicate predefined aging-related functional annotation categories used for visualization. **(e)** PPI and functional module analysis of 128 nominally altered aging-related proteins selected for exploratory analysis. Network modules were generated using Metascape based on interaction and functional enrichment analyses. Node colors indicate proteins belonging to the same functional module, and edges indicate known or predicted protein-protein associations. Representative modules included interferon alpha/beta signaling, activation of NF-κB in B cells, chromatin remodeling, cellular response to chemical stress, and meiosis. Functional enrichment significance was evaluated using the statistical framework implemented in Metascape, and enrichment *P* values are shown as log10(*P*) values in the module labels.

PBMCs were collected from a self-established cohort comprising 82 patients with SLE_S, 48 patients with SLE_A, and 90 HCs. The study groups were broadly comparable in age and sex as expected between HC, SLE_S and SLE_A groups; detailed clinical characteristics are provided in **Supplementary Table 2**. To obtain sufficient protein input for phosphopeptide enrichment and subsequent proteomic and phosphoproteomic analyses, PBMC proteins from approximately six participants within the same clinical group were combined to generate one analytical pool. This procedure resulted in 14 SLE_S pools, 7 SLE_A pools, and 15 HC pools, each containing 1 mg of total protein. These analytical pools, rather than individual participants, constituted the independent analytical units. Using 4D label-free quantification, we identified 2,602 proteins and 8,329 phosphorylation sites across 2,787 proteins (9) (**Figure 1A**, **Supplementary Tables 3**, **4**).

We performed GSEA on differentially expressed proteins between SLE patients and HCs. GO-BP analysis identified DNA repair and double-strand break repair as the top enriched pathways (NES > 1.5, p < 0.05), which are involved in cellular stress responses and have been reported in aging-related molecular studies (19). HALLMARK analysis further indicated activation of pathways, including interferon gamma response, interferon alpha response (20), and the G2M checkpoint (21), representing canonical immune activation and cell-cycle regulatory programs in SLE. In contrast, pathways including xenobiotic metabolism (22), PI3K-AKT-mTOR signaling (23), reactive oxygen species (24), and fatty acid metabolism (22) were downregulated (**Figure 1B**). Overall, these results suggest that SLE-associated proteomic alterations reflect immune and stress-response reprogramming, which partially overlaps with molecular signatures described in aging-related contexts.

To further delineate aging-related alterations, differential proteins were mapped to aging-related biological categories (**Supplementary Table 1**) (22). GSEA revealed enrichment across six aging-related dimensions in SLE, including genomic instability, epigenetic alterations, intercellular communication, cell proliferation, mitochondrial function, and nutrient sensing (**Figures 1C, D**) (22). A total of 128 aging-related genes were identified across these dimensions, providing a multidimensional view of SLE-associated proteomic alterations across aging-related biological processes.

To assess the cell-type context of the bulk PBMC proteomic findings, we compared the direction of protein abundance changes with cell-type-specific differential expression patterns from in-house scRNA-seq cohort (GSE158263). Concordant transcript-protein changes were observed across multiple T-cell, B-cell, monocyte, dendritic-cell, and NK-cell subsets in the SLE_S versus HC, SLE_A versus HC, and SLE_A versus SLE_S comparisons (**Supplementary Figure 1**). These results provide partial cell-type-resolved support for a subset of the bulk proteomic alterations, although they do not distinguish cell-intrinsic regulation from compositional effects.

We next constructed a PPI network of the 128 proteins and their closely associated subnetworks using the Metascape platform. Network analysis revealed that proteins involved in interferon alpha/beta signaling exhibited the highest connectivity (log10(P) = −11.2). In addition, protein groups related to NF-κB activation in B cells, cellular response to chemical stress, chromatin remodeling, and meiosis also showed strong interactions (**Figure 1E**).

### 3.2 Phosphoproteomic remodeling of aging-related signaling networks in SLE

**Figure 2:**
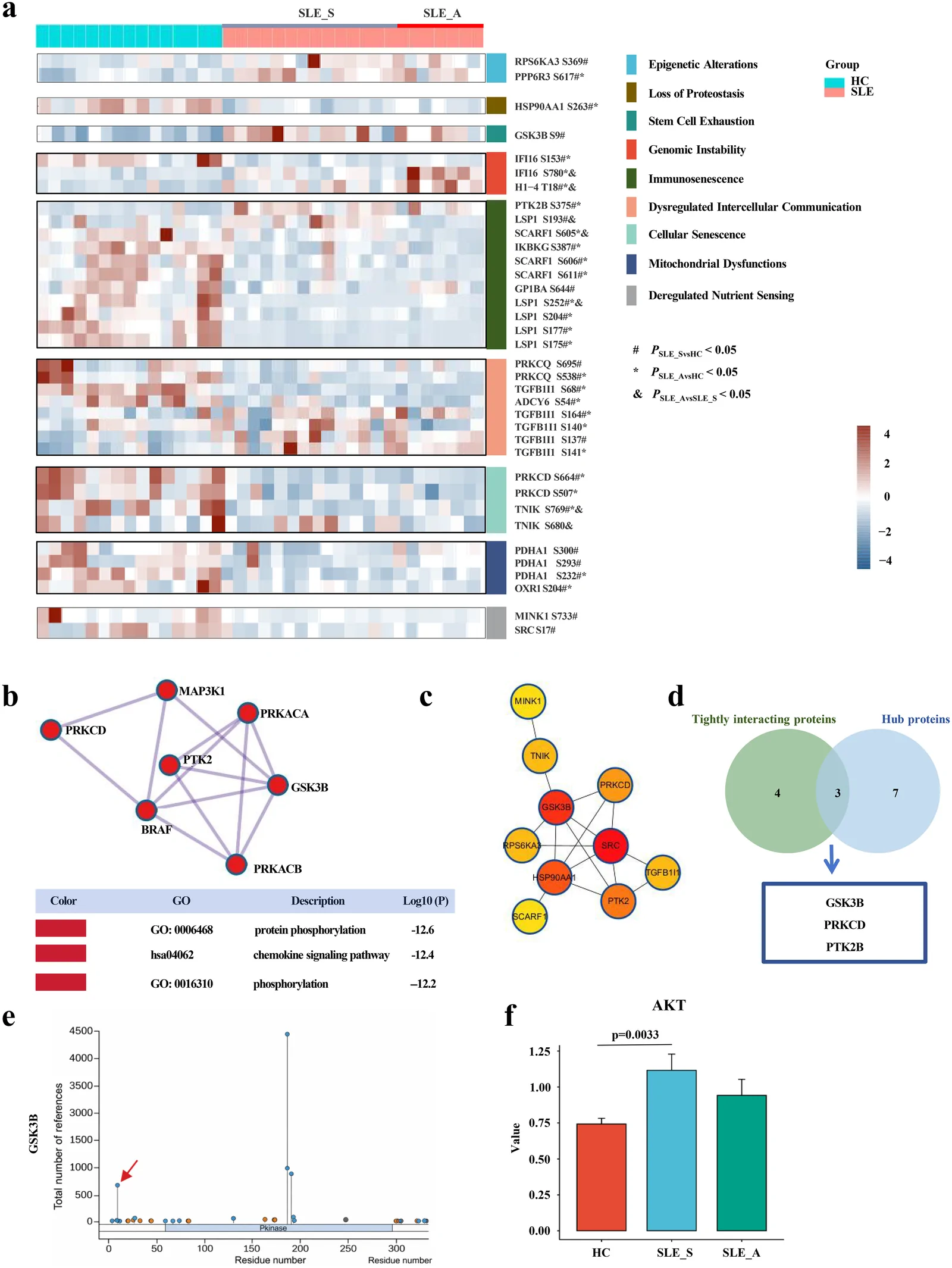
Phosphorylation profiling of aging-related proteins in SLE immune cells. **(a)** Heatmap showing 36 nominally altered phosphorylation sites selected for exploratory visualization in PBMCs from 14 SLE_S analytical pools, 7 SLE_A analytical pools, and 15 HC analytical pools. The source cohort comprised 82 patients with SLE_S, 48 patients with SLE_A, and 90 HCs, with PBMC proteins from approximately six participants within the same clinical group combined into each analytical pool. These analytical pools, rather than individual participants, constituted the independent analytical units. Differential phosphorylation was assessed using two-sample, two-sided Student’s t test at the analytical-pool level, and phosphorylation sites with nominal *P* < 0.05 were selected for exploratory visualization. To address multiple testing, Benjamin-Hochberg-adjusted *P* values were additionally calculated using 8,329 quantifiable phosphorylation sites as the multiple-testing background and are provided in the corresponding **Supplementary Table 4**. Only phosphorylation sites meeting the prespecified FDR threshold were considered robust after multiple-testing correction. Effect sizes are reported as log2 fold changes. Rows represent phosphorylation sites and columns represent independent analytical pools rather than individual participants. Red and blue colors indicate relatively increased and decreased phosphorylation abundance, respectively, based on row-scaled values. The top annotation denotes sample groups. The colored side bars indicate predefined aging-related functional annotation categories used for visualization. **(b)** PPI network of proteins containing the 36 nominally altered phosphorylation sites. The network was constructed using STRING/Cytoscape based on known or predicted protein-protein associations. Nodes represent proteins, and edges represent PPIs. **(c)** Candidate hub proteins identified from the PPI network using cytoHubba analysis. Nodes represent proteins, and node ranking reflects the hub score calculated by cytoHubba. GSK3B, SRC, and HSP90AA1 were among the top-ranked proteins. **(d)** Overlap analysis between tightly interacting proteins and candidate hub proteins identified GSK3B, PRKCD, and PTK2 as shared candidate nodes potentially involved in the phosphorylation-associated network in SLE. Numbers in the Venn diagram indicate the number of overlapping or non-overlapping proteins in each group. **(e)** PhosphoSitePlus annotation of phosphorylation sites on GSK3B. The x-axis indicates amino acid residue positions, and the y-axis indicates the number of reported records for each phosphorylation site in PhosphoSitePlus. The red arrow marks S9, one of the most frequently reported phosphorylation sites on GSK3B. **(f)** Protein abundance of AKT1, an upstream regulator of GSK3B S9 phosphorylation, in 15 HC analytical pools, 14 SLE_S analytical pools, and 7 SLE_A analytical pools. Data are shown as mean ± SEM, and each dot represents one independent analytical pool. Group differences were assessed using two-sample, two-sided Student’s t test at the analytical-pool level. The displayed *P* value is a nominal, unadjusted *P* value and should be interpreted as exploratory.

We next examined phosphorylation site changes between SLE patients and HCs. A total of 36 phosphorylation sites in aging-related proteins showed differential abundance, spanning multiple aging-related dimensions (**Figure 2A**).

Among these, phosphorylation sites in IFI16 (S153, S780) and PRKCD (S507, S664) showed nominally significant differential abundance and were retained as exploratory features. While these proteins have been previously implicated in immune regulation, the functional relevance of specific phosphorylation sites remains unclear, representing potentially novel observations. Additional differentially phosphorylated sites were identified in proteins with less well-characterized roles in SLE, including LSP1, SCARF1, and TNIK.

To further characterize the biological processes associated with proteins exhibiting altered phosphorylation in **Figure 2A**, we constructed a PPI network and analyzed closely interacting modules. Network analysis indicated that these proteins were predominantly enriched in kinase-related processes involved in protein phosphorylation (log10(P) = −12.6) (**Figure 2B**).

To identify central nodes within the network, hub analysis was performed using the MCC algorithm in Cytoscape. GSK3β (GSK3B), Src (SRC), and Hsp90α (HSP90AA1) showed the highest scores (**Figure 2C**), indicating high network connectivity.

We further intersected hub proteins with tightly interacting network components and identified GSK3β (GSK3B), PKCδ (PRKCD), and FAK (PTK2) as overlapping candidates (**Figure 2D**). These proteins exhibited both strong interaction connectivity and high hub scores, suggesting potential central positions within the phosphorylation-associated network in SLE.

Among these candidates, GSK3β (GSK3B) showed the highest hub score. Phosphoproteomic analysis revealed increased phosphorylation at the S9 site of GSK3β in SLE. Notably, PhosphoSitePlus annotation indicated that S9 is a well-characterized phosphorylation site with extensive prior evidence, supporting its potential regulatory significance (**Figure 2E**).

Previous studies have reported that AKT1 can phosphorylate GSK3β at S9 (25). Consistent with these findings, we observed increased AKT1 protein levels in SLE_S compared with HCs (**Figure 2F**), suggesting a potential association between AKT1 activity and GSK3β S9 phosphorylation in SLE.

### 3.3 Disease-status- and activity-associated proteomic and phosphoproteomic patterns in SLE

**Figure 3:**
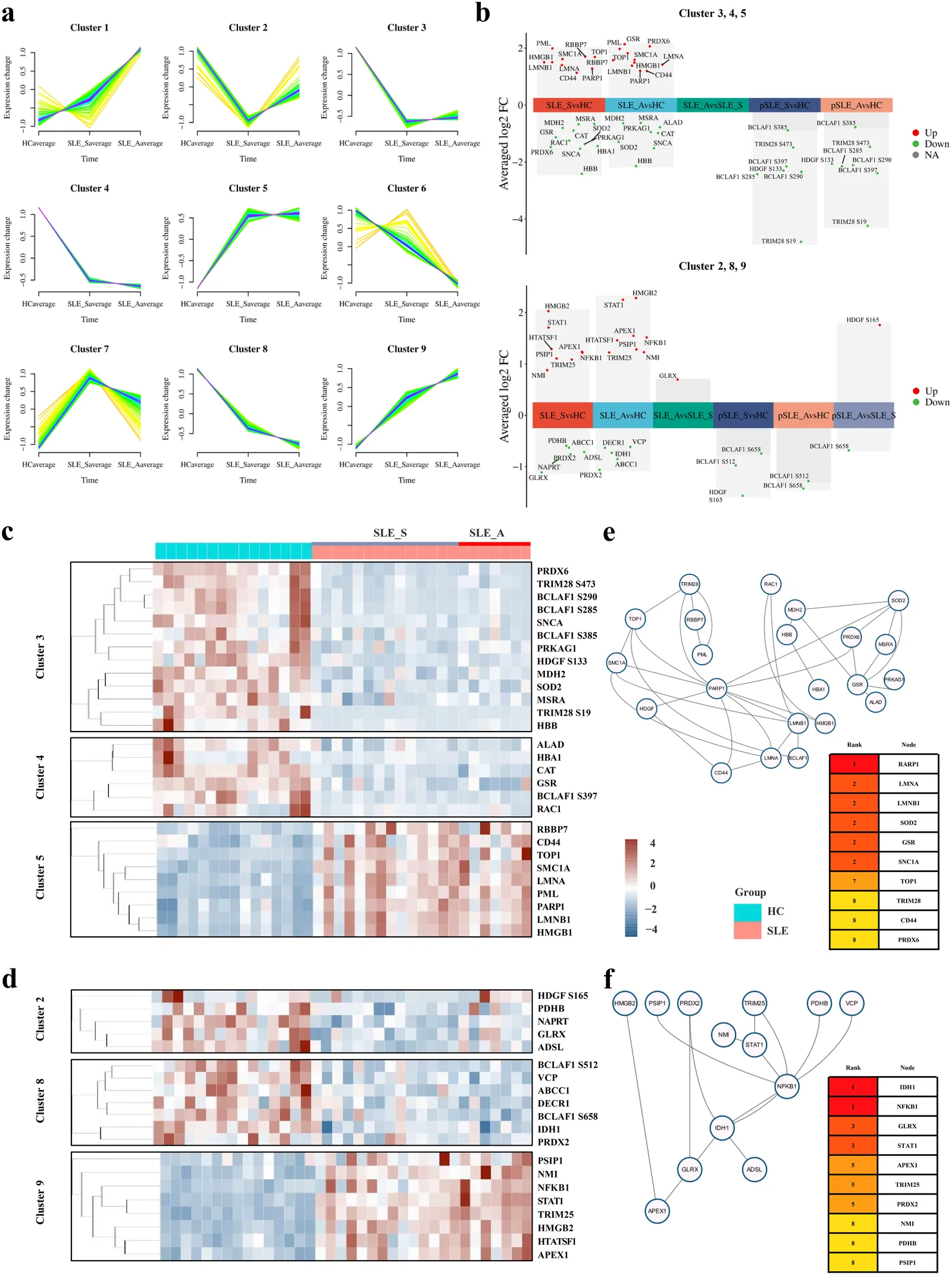
Disease-status- and disease-activity-associated proteomic and phosphoproteomic signatures in SLE. **(a)** Cluster analysis of exploratory proteomic and phosphoproteomic features identified nine group-level abundance patterns across 15 HC analytical pools, 14 SLE_S analytical pools, and 7 SLE_A analytical pools. These analytical pools, rather than individual participants, constituted the independent analytical units. Clusters 3, 4, and 5 were interpreted as disease-status-associated clusters, whereas clusters 2, 8, and 9 were interpreted as disease-activity-associated clusters based on their relative abundance patterns across HC, SLE_S, and SLE_A groups. Each colored line represents the scaled group-level abundance pattern of one protein or phosphorylation site across the three groups. Because the data were cross-sectional, these patterns were not interpreted as longitudinal or temporal trajectories. **(b)** Volcano plots showing nominally altered proteins and phosphorylation sites in disease-status-associated clusters (clusters 3, 4, and 5) and disease-activity-associated clusters (clusters 2, 8, and 9). In the volcano plots, red dots indicate increased features, and green dots indicate decreased features. The displayed features were selected for exploratory analysis using the nominal *P*-value thresholds defined in the Methods; corresponding FDR-adjusted *P* values are provided in **Supplementary Table 3** and **4**. **(c, d)** Heatmaps showing the abundance patterns of proteins and phosphorylation sites within disease-status-associated clusters (clusters 3, 4, and 5) and disease-activity-associated clusters (clusters 2, 8, and 9) across independent analytical pools. Rows represent proteins or phosphorylation sites, and columns represent independent analytical pools rather than individual participants. Red and blue colors indicate relatively increased and decreased abundance, respectively, based on row-scaled values. The top annotation indicates sample groups, with cyan representing HCs and red representing SLE samples. **(e, f)** PPI networks and hub-protein analysis of features included in the disease-status-associated and disease-activity-associated cluster groups. Networks were constructed based on known or predicted PPIs, and candidate hub proteins were ranked using CytoHubba. Nodes represent proteins, and edges represent PPIs. The accompanying ranked tables show the top candidate hub proteins identified in each network. These network and hub analyses were exploratory and do not establish functional or causal regulatory relationships.

To distinguish proteins potentially associated with SLE disease status from those associated with disease activity, we analyzed the expression patterns of 128 aging-related proteins and 36 phosphorylation sites across HC, SLE_S, and SLE_A groups. We evaluated solutions with 8–11 clusters. The nine-cluster solution was selected because it preserved the major reproducible expression trajectories while avoiding excessive subdivision of biologically similar patterns observed in the 10- and 11-cluster solutions (**Figure 3A**, **Supplementary Figure 2**).

Clusters 3, 4, and 5 showed differences between SLE_S and HC but remained relatively stable between SLE_A and SLE_S, suggesting potential associations with SLE disease status that were largely independent of activity level. In contrast, Clusters 2, 8, and 9 exhibited changes across SLE_S and SLE_A, indicating potential associations with disease activity. Clusters 1, 6, and 7 showed heterogeneous patterns and were not considered for further analysis (**Figure 3B**).

Proteins in Clusters 3–5 included factors associated with chromatin organization, genomic stability, and metabolic processes, such as HMGB1, RBBP7, SMC1A, MDH2, GSR, and SOD proteins (26–31). These molecular patterns may therefore reflect biological alterations associated with the presence of SLE rather than changes related to clinical activity.

Proteins in Clusters 2, 8, and 9 showed stepwise increases across the three clinical groups, including HMGB2, STAT1, APEX1, and NFKB1. These proteins have been previously implicated in inflammatory signaling, DNA repair, and transcriptional regulation (32–35), suggesting that these processes may vary according to SLE activity status.

We also identified a subset of proteins, including HDGF S165, PDHB, NAPRT, GLRX, and ADSL that showed decreased levels in SLE_S followed by partial restoration in SLE_A. These proteins are involved in redox regulation and metabolic processes (36–38), suggesting nonlinear metabolic alterations across the clinical groups.

PPI analysis was performed separately for proteins in Clusters 3–5 and Clusters 2, 8, and 9. Proteins in Clusters 3–5 showed network connectivity involving molecules such as PARP1, LMNA, LMNB1, SOD2, and GSR, which are associated with chromatin organization, nuclear structure, and metabolic processes (**Figure 3C**). In contrast, proteins in Clusters 2, 8, and 9 showed network connectivity involving immune-related regulators, including NFKB1, STAT1, APEX1, and PSIP1 (**Figure 3D**).

Hub analysis using CytoHubba identified PARP1, LMNA, and LMNB1 as top-ranked nodes within the disease-status-associated clusters (**Figure 3E**), whereas IDH1, NFKB1, and GLRX were among the top-ranked nodes within the disease-activity-associated clusters (**Figure 3F**). Several of these proteins, including NFKB1 and PARP1, have been previously implicated in immune regulation and DNA repair processes, whereas others remain less well characterized. Because these analyses were based on cross-sectional clinical groups, the observed patterns should be interpreted as associations with disease status or activity rather than as evidence of disease initiation or longitudinal progression.

### 3.4 Multi-omics integration identifies putative transcriptional regulatory networks of aging-related genes in SLE

To investigate the upstream regulatory landscape of aging-related genes in SLE, we integrated transcriptomic, proteomic, and phosphoproteomic data to construct a putative and exploratory transcriptional regulatory network.

**Figure 4:**
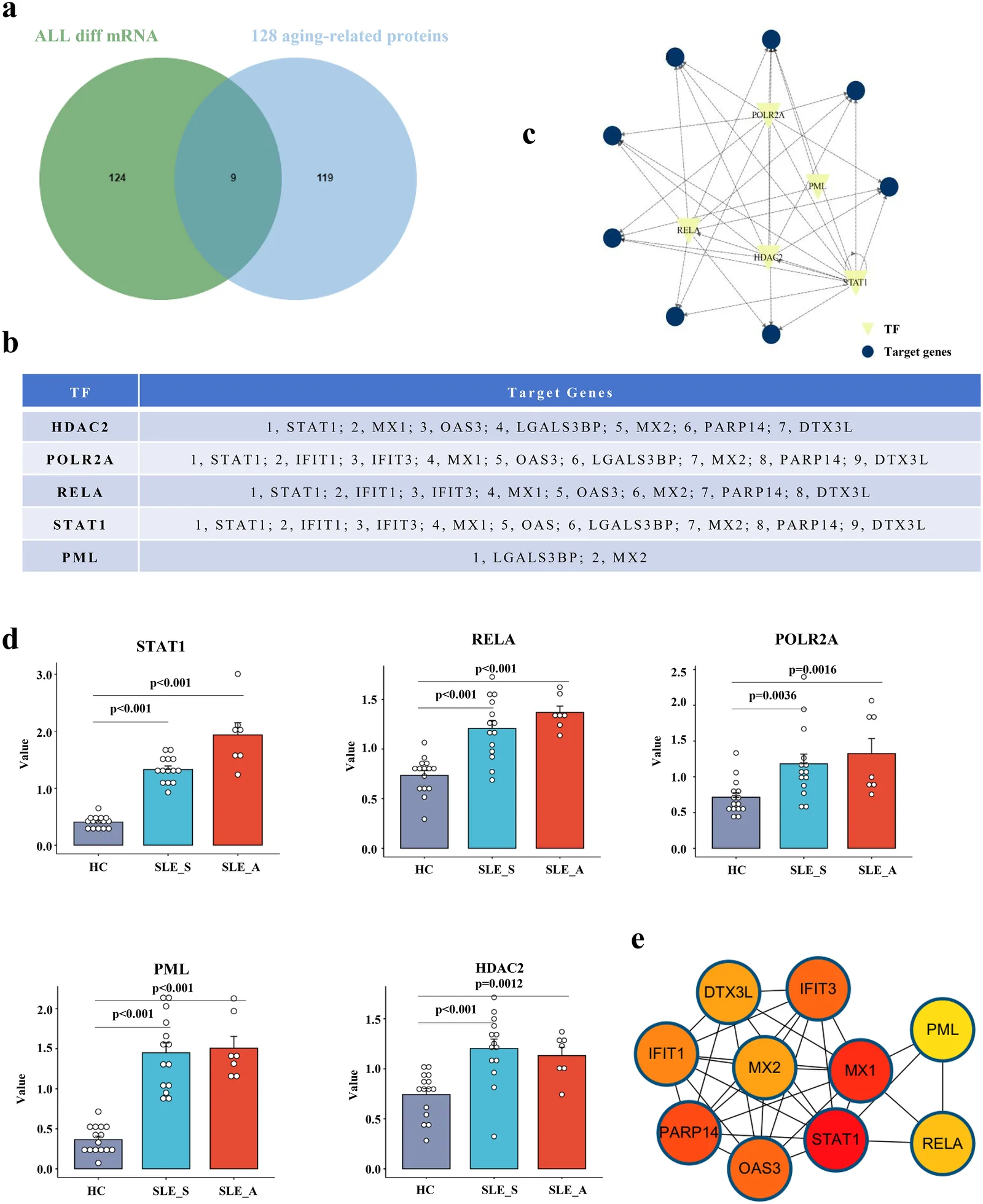
Putative TF regulatory networks of aging-related genes in SLE. **(a)** Venn diagram showing the overlap between differentially expressed mRNAs and the 128 nominally altered aging-related proteins selected for exploratory analysis, identifying nine cross-omics concordant genes across transcriptomic and proteomic datasets. Differentially expressed mRNAs were defined as described in the Methods. The proteomic features were identified at the analytical-pool level, and corresponding FDR-adjusted *P* values are provided in **Supplementary Table 3**. **(b)** Candidate upstream TFs and their predicted target genes were annotated using the hTFtarget database. These TF-target relationships were used for exploratory regulatory inference and should not be interpreted as direct functional validation. **(c)** TF-target gene regulatory network showing the relationships between predicted upstream TFs and the nine overlapping aging-related genes. Yellow triangles represent TFs, and blue circles represent target genes. Edges indicate database-derived putative regulatory relationships rather than experimentally confirmed interactions in the present study. **(d)** Protein abundance levels of the candidate TFs STAT1, RELA, POLR2A, PML, and HDAC2 across 15 HC analytical pools, 14 SLE_S analytical pools, and 7 SLE_A analytical pools. Bars indicate mean ± SEM, each dot represents one independent analytical pool rather than an individual participant. Group comparisons were performed using two-sample, two-sided Student’s t-tests at the analytical-pool level. The displayed *P* values nominal, unadjusted *P* values and should be interpreted as exploratory. **(e)** Interaction network integrating candidate TFs and aging-related target genes. Nodes represent TFs or target genes, and edges represent predicted regulatory or interaction relationships. Node color intensity represents the hub-protein ranking, with redder nodes indicating higher-ranked hub proteins. This network was generated through computational integration and topological analysis and does not establish direct or causal regulatory relationships.

A total of 1,461 SLE samples and 198 HCs were included in the transcriptomic analysis. Differential expression analysis identified 133 genes (fold change > 1.5, adjusted *P* < 0.05). The intersection of these genes with 128 differentially abundant aging-related proteins yielded nine overlapping targets, including STAT1, IFIT1, IFIT3, MX1, OAS3, LGALS3BP, MX2, PARP14, and DTX3L (**Figure 4A**). Notably, these genes were therefore considered a limited set of cross-omics concordant candidates, rather than evidence of broad transcriptomic-proteomic agreement or comprehensive external validation.

To explore upstream regulatory mechanisms, we performed TF analysis and identified multiple candidate regulators of these aging-related genes. Among these, STAT1, RELA, POLR2A, PML, and HDAC2 showed increased protein abundance in SLE compared with HCs, suggesting their potential involvement in the inferred regulatory network (**Figures 4B–D**).

We further constructed a PPI network linking these TFs and downstream targets, followed by hub analysis to identify topologically prominent nodes. MX1, STAT1, PARP14, OAS3, and IFIT3 were identified among the top-ranked nodes (**Figure 4E**). These network relationships were primarily derived from computational integration and bioinformatic inference and therefore should be interpreted as hypothesis-generating rather than functionally validated regulatory interactions.

Several of these proteins, including STAT1 and MX1, have been extensively implicated in SLE pathogenesis, particularly in interferon signaling and immune activation (39–41). In contrast, others, such as PARP14, OAS3, and IFIT3, are less well characterized in this context.

### 3.5 Phosphoproteomics-inferred kinase activity highlights disease-status-associated and disease-activity-associated signaling networks in SLE

To explore upstream kinase regulators associated with these differentially phosphorylated aging-related proteins, we inferred kinase activity changes across comparison groups based on phosphoproteomic data (**Supplementary Table 5**).

**Figure 5:**
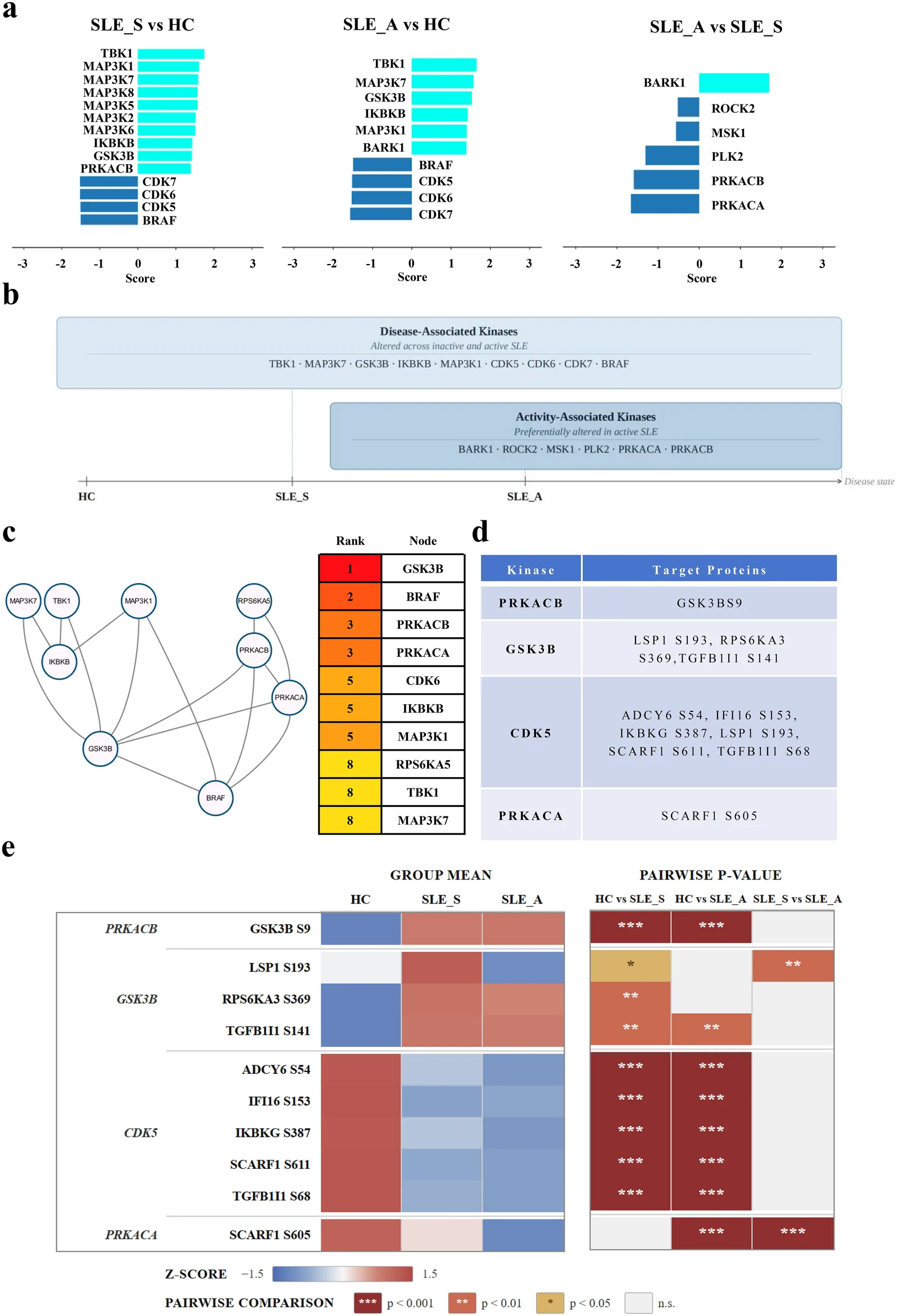
Disease-status- and disease-activity-associated kinase activity and signaling networks in SLE. **(a)** Predicted kinase activity across pairwise comparisons, including SLE_S versus HC, SLE_A versus HC, and SLE_A versus SLE_S. Bars indicate kinase activity scores; positive values indicate predicted activation and negative values indicate predicted inhibition. Kinase activity was inferred computationally from phosphoproteomic data obtained from 15 HC analytical pools, 14 SLE_S analytical pools, and 7 SLE_A analytical pools. **(b)** Classification of kinases into disease-status-associated and disease-activity-associated groups according to their differential activity patterns. Disease-status-associated kinases were consistently altered in both SLE_S versus HC and SLE_A versus HC comparisons, whereas disease-activity-associated kinases were mainly altered in the SLE_A versus SLE_S comparison. Because the data were cross-sectional, these classifications indicate associations with disease status or activity rather than temporal changes. **(c)** PPI network of candidate kinases, highlighting GSK-3β/GSK3B, BRAF, and PRKACB as potential central nodes. Nodes represent kinases and edges represent known or predicted PPIs. **(d)** Putative Kinase-substrate relationships identified using PhosphoSitePlus. The network and centrality analysis were exploratory and do not establish functional or causal kinase relationships. **(e)** Heatmap showing the phosphorylation levels of predicted kinase substrates across 15 HC analytical pools, 14 SLE_S analytical pools, and 7 SLE_A analytical pools. Values are shown as row-scaled Z-scores based on phosphosite abundance. Columns represent independent analytical pools rather than individual participants. Pairwise differences among groups were assessed using two-sample, two-sided Student’s t-tests at the analytical-pool level. Multiple testing was evaluated using the Benjamini-Hochberg method in the corresponding differential phosphorylation analysis; the significance symbols displayed in the heatmap represent nominal, unadjusted pairwise *P* values and should be interpreted as exploratory. Corresponding FDR-adjusted *P* values are provided in **Supplementary Table 4**, and only results meeting the prespecified FDR threshold were considered robust after multiple-testing correction. *P < 0.05, **P < 0.01, ***P < 0.001; n.s., not significant.

Several kinases, including TBK1, MAP3K1, MAP3K7, IKBKB, GSK3B, BRAF, and CDK5/6/7, showed altered predicted activity in both SLE_S vs HC and SLE_A vs HC comparisons, suggesting potential roles in disease-associated signaling. In contrast, kinases such as BARK1, ROCK2, MSK1, PLK2, PRKACA, and PRKACB were more prominently altered in the SLE_A vs SLE_S comparison, indicating potential associations with disease activity (**Figures 5A, B**).

Several of these kinases, including TBK1, GSK3B, and IKBKB, have been extensively implicated in inflammatory signaling and immune regulation (42–45), whereas others, such as BARK1, ROCK2, MSK1, PLK2, and PRKACA, remain less characterized in SLE.

Network analysis indicated that these kinases were interconnected, with GSK3B, BRAF, and PRKACB emerging as topologically prominent nodes within the kinase interaction network (**Figure 5C**).

To further explore potential downstream substrates, kinase-substrate relationships were predicted using the PhosphoSitePlus tool. These analyses suggested that multiple kinases may converge on proteins involved in immune signaling and cytoskeletal regulation. For example, predicted targets of GSK3B included LSP1 (S193), RPS6KA3 (S369), and TGFB1I1 (S141), whereas CDK5 was predicted to target proteins such as ADCY6 (S54), IFI16 (S153), and IKBKG (S387). In addition, PRKACA was predicted to target LSP1 (S177), SCARF1 (S605/S606), and TGFB1I1 (S68). These predictions provide a resource for potential kinase-substrate interactions that may be relevant to SLE-associated signaling processes (**Figures 5D, E**).

Together, these observations provide an integrated view of putative kinase-associated regulation of these differentially phosphorylated aging-related proteins across SLE disease stages, although further experimental validation is required to confirm the inferred regulatory relationships.

## 4 Discussion

**Figure 6:**
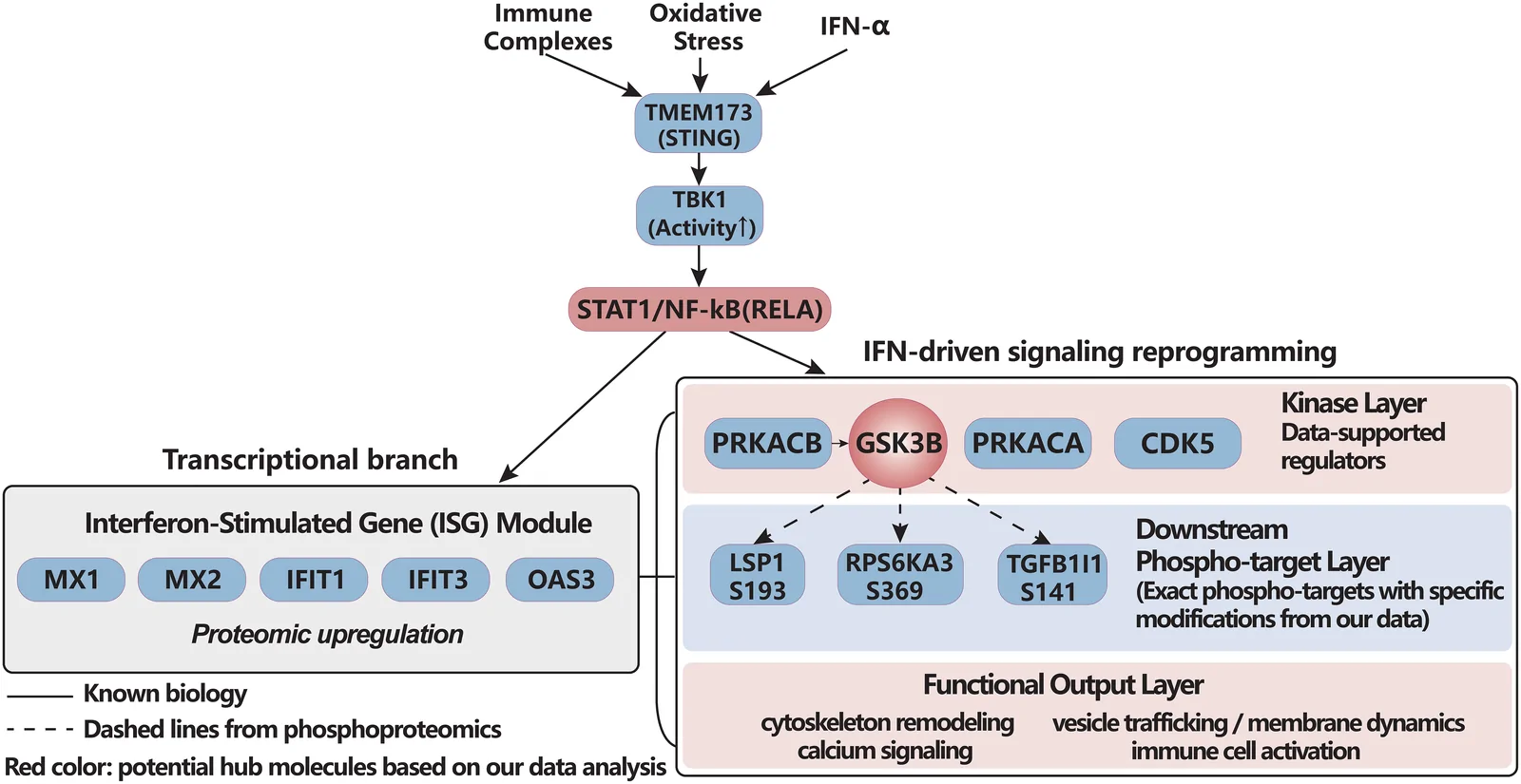
Multi-omics-integrated model of aging-related molecular alterations and interferon-associated regulatory signaling in SLE. Multi-omics integration of aging-related nominally altered proteins and phosphorylation sites, and public transcriptomic data suggested that SLE-associated aging-related molecular alterations are linked to interferon-associated transcriptional and kinase signaling programs. The model highlights a putative STING-TBK1-associated signaling axis connected to STAT1/NF-κB-related transcriptional programs and interferon-stimulated gene (ISG) expression. Phosphoproteomic analysis further nominated a GSK3B-centered candidate kinase network associated with downstream phosphorylation events on aging-related proteins. Solid lines indicate literature-supported relationships, whereas dashed lines represent inferred or computationally predicted relationships. This model is intended as a hypothesis-generating summary rather than a validated regulatory mechanism.

In this study, we integrated proteomic, phosphoproteomic, and transcriptomic data to characterize SLE-associated molecular alterations that overlap with aging-related biological processes. The identified signatures were dominated by inflammatory and interferon-associated pathways and converged on regulatory networks involving STAT1, RELA, TBK1, IKBKB, and GSK3B. These findings do not directly demonstrate accelerated immune aging or inflammaging. Rather, they indicate that SLE-associated immune activation shares molecular features with several biological processes implicated in aging and that these features are organized around interconnected transcriptional and kinase networks (**Figure 6**).

Inflammaging refers to chronic, low-grade systemic inflammation associated with aging and is characterized by sustained activation of innate immune pathways, altered cytokine production, and remodeling of immune and metabolic homeostasis (46). Although type I interferon and NF-κB signaling may overlap with pathways implicated in inflammaging, their activation in SLE more directly reflects disease-specific immune dysregulation, including nucleic acid sensing, immune-complex-driven inflammation, and chronic interferon pathway activation (47, 48). Therefore, the interferon- and NF-κB-related signatures observed in this study should be interpreted as SLE-associated inflammatory programs that overlap with aging-related molecular features, rather than as direct evidence of inflammaging or accelerated immune aging.

Notably, the hallmarks of aging framework was updated by López-Otín et al. in 2023, expanding the original model to 12 hallmarks by incorporating disabled macroautophagy, chronic inflammation, and dysbiosis (X). We did not reclassify our findings strictly according to the updated framework because the present analysis was based on predefined aging-related gene resources and PBMC proteomic and phosphoproteomic data, which do not comprehensively capture all components of the 2023 framework, particularly dysbiosis and organism-level aging processes. Accordingly, our findings indicate molecular overlap between SLE-associated inflammatory, metabolic, and stress-response alterations and aging-related biology, but do not establish accelerated biological aging or activation of specific aging hallmarks in SLE.

Several previous studies have reported SLE-associated alterations in biological processes that also occur during aging, including interferon signaling, mitochondrial dysfunction, epigenetic dysregulation, and chronic inflammatory activation (3–5). Our findings are generally consistent with these observations. Compared with previous studies that primarily focused on individual molecular layers, the current study integrated proteomic, phosphoproteomic, and transcriptomic data using aging-related biological processes as an interpretative framework and incorporated analyses across inactive and active SLE. In addition, the inferred TF and kinase regulatory networks provided a systems-level view of interconnected signaling alterations linking SLE-associated immune dysregulation with aging-related molecular processes.

Through phosphoproteomic analysis, we identified alterations in specific phosphorylation sites associated with aging in SLE. For example, increased phosphorylation was observed at the S153 and S780 sites of IFI16 and the S605 site of SCARF1. Previous studies have reported that both IFI16 and SCARF1 are involved in the pathogenesis of SLE. IFI16 is associated with DNA damage and genomic instability (49–51), while SCARF1 is closely linked to immune imbalance (52, 53).

Notably, we identified phosphorylation changes in proteins such as LSP1 (S193, S252), TGFB1I1 (S68, S137), and PDHA1 (S300, S293), whose roles in SLE remain poorly characterized. These findings suggest that phosphorylation-mediated regulation of these proteins may represent previously underexplored mechanisms in SLE pathogenesis.

In SLE, our phosphoproteomic analysis detected increased phosphorylation of GSK3β at Ser9, a modification generally associated with reduced kinase activity downstream of AKT1 activation. By contrast, kinase-substrate enrichment analysis suggested preserved or increased GSK3β-related activity. This apparent discrepancy may reflect the complex regulation of GSK3β, which is influenced not only by Ser9 phosphorylation but also by other post-translational modifications, subcellular localization, substrate availability, and signaling crosstalk. Alternative regulatory mechanisms, cellular heterogeneity within bulk PBMCs, and limitations of the computational activity-inference model may therefore contribute to the discordant findings. Accordingly, the inferred role of GSK3β should be regarded as hypothesis-generating, and direct functional validation is required to establish its actual activity and biological significance in SLE.

Despite the integrative multi-omics design, several limitations should be acknowledged. First, this cross-sectional study identified disease-status- and disease-activity-associated differences across HC, SLE_S, and SLE_A, but these patterns do not represent true longitudinal dynamics and should not be interpreted as direct evidence of inflammaging or accelerated immune aging. Second, although the analyzed molecules were curated from aging-related gene resources, the observed alterations may also reflect SLE-related inflammation, interferon activation, disease activity, treatment exposure, and PBMC compositional changes. Because proteomic and phosphoproteomic measurements were generated from pooled PBMC samples and several clinical variables, including menopausal status, disease duration, and complete treatment exposure, were unavailable, individual-level covariate adjustment and age-interaction analyses could not be reliably performed. Third, bulk PBMC profiling cannot resolve cell-type-specific molecular changes. Although scRNA-seq-based comparison provided supportive cell-type-resolved context for a subset of findings, sorted-cell proteomic or phosphoproteomic validation is still required. Fourth, the public transcriptomic integration was exploratory and based on a pooled, batch-corrected analysis rather than a formal study-level meta-analysis, with only limited transcriptome-proteome overlap. Finally, the inferred kinase and transcription factor networks, including the complex GSK3B-related findings, should be considered hypothesis-generating and require functional validation in independent cohorts.

## 5 Conclusions

In conclusion, this integrative multi-omics analysis identified inflammatory and interferon-dominated molecular alterations in SLE PBMCs that overlap with multiple aging-related molecular resources. These alterations converged on interconnected transcriptional and kinase networks involving STAT1, RELA, TBK1, IKBKB, and GSK3B. The findings provide a systems-level and hypothesis-generating framework for investigating the molecular intersection between chronic immune activation and aging-related biology in SLE. However, they should not be interpreted as direct evidence of accelerated immune aging or inflammaging, which will require longitudinal, cell-type-resolved, and mechanistic validation in independent cohorts.

## Data Availability

Publicly available datasets were analyzed in this study. This data can be found here: The phosphoproteomics datasets generated in this study have been deposited in the ProteomeXchange Consortium under accession numbers PXD025559 and PXD025399, respectively. MS/MS spectra can be visualized via MS-Viewer at the following URL: https://msviewer.ucsf.edu/prospector/cgi-bin/mssearch.cgi?report_title=MS-Viewer&search_key=wvw7pyeqyq&search_name=msviewer.
