# Supplementary material for: Integrated multi-omics profiling identifies aging-related molecular signatures and convergent interferon signaling in systemic lupus erythematosus
Source: Front Immunol. 2026 Aug 19;17:1861904. doi: 10.3389/fimmu.2026.1861904 (PMC13534008; doi:10.3389/fimmu.2026.1861904)
Supplement: Supplementary file 1 [file DataSheet1.zip › Supplementary Materials/Supplementary Figure S1.docx]

**Supplementary Figure S1. Cell-Type Distribution of Concordant Proteomic and Single-Cell Transcriptomic Alterations in SLE.** Differentially abundant proteins identified in bulk PBMC proteomic analyses were compared with cell-type-specific differentially expressed genes derived from the scRNA-seq dataset GSE158263. Results are shown for the SLE_S versus HC, SLE_A versus HC, and SLE_A versus SLE_S comparisons. Only genes exhibiting concordant directions of change at both the protein and transcript levels are displayed. Red circles indicate concordant upregulation, whereas blue circles indicate concordant downregulation. Columns represent annotated immune-cell subsets, and rows represent concordant genes. Blank cells indicate that no concordant transcript-protein alteration was detected for the corresponding gene and cell type. SLE_S, inactive SLE; SLE_A, active SLE; HC, healthy control.
