# Supplementary material for: Integrated multi-omics profiling identifies aging-related molecular signatures and convergent interferon signaling in systemic lupus erythematosus
Source: Front Immunol. 2026 Aug 19;17:1861904. doi: 10.3389/fimmu.2026.1861904 (PMC13534008; doi:10.3389/fimmu.2026.1861904)
Supplement: Supplementary file 1 [file DataSheet1.zip › Supplementary Materials/Supplementary Figure S2.docx]

**Supplementary Figure S2.** **Robustness Assessment of Mfuzz Clustering using Alternative Cluster Numbers.** Mfuzz clustering was repeated using alternative cluster solutions to evaluate the stability of the identified temporal expression patterns across HC, SLE_S, and SLE_A. (A) Eight-cluster solution. (B) Ten-cluster solution. (C) Eleven-cluster solution. Each panel shows the expression patterns of proteins assigned to the corresponding cluster, with line color indicating cluster membership values. Across the alternative solutions, the major temporal expression patterns were broadly preserved. Reducing the number of clusters mainly resulted in the merging of related trajectories, whereas increasing the number of clusters predominantly subdivided broader patterns without generating fundamentally different trends. These results support the qualitative robustness of the nine-cluster solution used in the main analysis. SLE_S, inactive SLE; SLE_A, active SLE; HC, healthy control.
