# Supplementary material for: Integrated multi-omics profiling identifies aging-related molecular signatures and convergent interferon signaling in systemic lupus erythematosus
Source: Front Immunol. 2026 Aug 19;17:1861904. doi: 10.3389/fimmu.2026.1861904 (PMC13534008; doi:10.3389/fimmu.2026.1861904)
Supplement: Supplementary file 1 [file DataSheet1.zip › Supplementary Materials/Supplementary Materials.docx]

**01_Transcriptomic_Data_Integration_and_ComBat_Batch_Correction.R**

\# Load required packages

library(limma)

library(sva)

outFile <- "merge.txt" # Output file

\# Set working directory

setwd("G:\\136machine-learning-biomarker\\05.sva\\sJIA&childrenSLE")

\# Retrieve all files ending with ".txt" in the working directory

files <- dir()

files <- grep("txt$", files, value = TRUE)

geneList <- list()

\# Read gene information from all text files and store it in geneList

for (file in files) {

if (file == outFile) {

next

}

rt <- read.table(

file,

header = TRUE,

sep = "\t",

check.names = FALSE

)

geneNames <- as.vector(rt[, 1]) # Extract gene names

uniqGene <- unique(geneNames)	# Retain unique gene names

header <- unlist(strsplit(file, "\\.|\\-"))

geneList[[header[1]]] <- uniqGene

}

\# Identify genes shared across all datasets

interGenes <- Reduce(intersect, geneList)

\# Initialize objects for data merging and batch annotation

allTab <- data.frame()

batchType <- c()

for (i in 1:length(files)) {

inputFile <- files[i]

header <- unlist(strsplit(inputFile, "\\.|\\-"))

\# Read and organize the expression matrix

rt <- read.table(

inputFile,

header = TRUE,

sep = "\t",

check.names = FALSE

)

rt <- as.matrix(rt)

rownames(rt) <- rt[, 1]

exp <- rt[, 2:ncol(rt)]

dimnames(exp) <- list(rownames(exp), colnames(exp))

data <- matrix(

as.numeric(as.matrix(exp)),

nrow = nrow(exp),

dimnames = dimnames(exp)

)

\# Collapse duplicate gene identifiers by averaging expression values

rt <- avereps(data)

\# Determine whether log2 transformation is required

qx <- as.numeric(

quantile(

rt,

c(0, 0.25, 0.5, 0.75, 0.99, 1.0),

na.rm = TRUE

)

)

LogC <- (

(qx[5] > 100) ||

((qx[6] - qx[1]) > 50 && qx[2] > 0)

)

if (LogC) {

rt[rt < 0] <- 0

rt <- log2(rt + 1)

}

\# Perform between-array normalization within each dataset

rt <- normalizeBetweenArrays(rt)

\# Merge datasets using the shared genes

if (i == 1) {

allTab <- rt[interGenes, ]

} else {

allTab <- cbind(allTab, rt[interGenes, ])

}

\# Record the batch label for each sample

batchType <- c(batchType, rep(i, ncol(rt)))

}

\# Correct dataset-associated batch effects using ComBat

outTab <- ComBat(

allTab,

batch = batchType,

par.prior = TRUE

)

\# Add sample names as the first row

outTab <- rbind(

geneNames = colnames(outTab),

outTab

)

\# Export the batch-corrected merged expression matrix

write.table(

outTab,

file = outFile,

sep = "\t",

quote = FALSE,

col.names = FALSE

)

**02_Limma_Differential_Expression_Analysis.R**

\# Load required packages

library(limma)

library(pheatmap)

inputFile <- "Ubnormalize.txt" # Input file

logFCfilter <- 0.585	# log2 fold-change threshold

adj.P.Val.Filter <- 1	# Adjusted P-value threshold

\# Set working directory

setwd("G:\\136machine-learning-biomarker\\06.diff\\COAD")

\# Read and organize the input expression matrix

rt <- read.table(

inputFile,

header = TRUE,

sep = "\t",

check.names = FALSE

)

rt <- as.matrix(rt)

rownames(rt) <- rt[, 1]

exp <- rt[, 2:ncol(rt)]

dimnames(exp) <- list(rownames(exp), colnames(exp))

data <- matrix(

as.numeric(as.matrix(exp)),

nrow = nrow(exp),

dimnames = dimnames(exp)

)

\# Collapse duplicate gene identifiers by averaging expression values

data <- avereps(data)

\# Retain genes with a mean expression value greater than zero

data <- data[rowMeans(data) > 0, ]

\# Read all files ending with "s1.txt" as control sample lists

sampleName1 <- c()

files <- dir()

files <- grep("s1.txt$", files, value = TRUE)

for (file in files) {

rt <- read.table(

file,

header = FALSE,

sep = "\t",

check.names = FALSE

)

geneNames <- as.vector(rt[, 1]) # Extract sample names

uniqGene <- unique(geneNames)	# Retain unique sample names

sampleName1 <- c(sampleName1, uniqGene)

}

\# Read all files ending with "s2.txt" as treatment sample lists

sampleName2 <- c()

files <- dir()

files <- grep("s2.txt$", files, value = TRUE)

for (file in files) {

rt <- read.table(

file,

header = FALSE,

sep = "\t",

check.names = FALSE

)

geneNames <- as.vector(rt[, 1]) # Extract sample names

uniqGene <- unique(geneNames)	# Retain unique sample names

sampleName2 <- c(sampleName2, uniqGene)

}

\# Extract control and treatment expression data

conData <- data[, sampleName1]

treatData <- data[, sampleName2]

data <- cbind(conData, treatData)

conNum <- ncol(conData)

treatNum <- ncol(treatData)

\# Perform differential expression analysis

Type <- c(

rep("con", conNum),

rep("treat", treatNum)

)

design <- model.matrix(~0 + factor(Type))

colnames(design) <- c("con", "treat")

fit <- lmFit(data, design)

cont.matrix <- makeContrasts(

treat - con,

levels = design

)

fit2 <- contrasts.fit(fit, cont.matrix)

fit2 <- eBayes(fit2)

\# Extract differential expression statistics with FDR adjustment

allDiff <- topTable(

fit2,

adjust = "fdr",

number = 200000

)

allDiffOut <- rbind(

id = colnames(allDiff),

allDiff

)

write.table(

allDiffOut,

file = "all.txt",

sep = "\t",

quote = FALSE,

col.names = FALSE

)

\# Export the expression matrix used for differential analysis

outData <- rbind(

id = paste0(colnames(data), "_", Type),

data

)

write.table(

outData,

file = "normalize.txt",

sep = "\t",

quote = FALSE,

col.names = FALSE

)

\# Filter and export differentially expressed genes

diffSig <- allDiff[

with(

allDiff,

abs(logFC) > logFCfilter &

adj.P.Val < adj.P.Val.Filter

),

]

diffSigOut <- rbind(

id = colnames(diffSig),

diffSig

)

write.table(

diffSigOut,

file = "diff.txt",

sep = "\t",

quote = FALSE,

col.names = FALSE

)

\# Export the expression matrix of differentially expressed genes

diffGeneExp <- data[row.names(diffSig), ]

diffGeneExpOut <- rbind(

id = paste0(colnames(diffGeneExp), "_", Type),

diffGeneExp

)

write.table(

diffGeneExpOut,

file = "diffGeneExp.txt",

sep = "\t",

quote = FALSE,

col.names = FALSE

)
