# Supplementary material for: Integrated multi-omics profiling identifies aging-related molecular signatures and convergent interferon signaling in systemic lupus erythematosus
Source: Front Immunol. 2026 Aug 19;17:1861904. doi: 10.3389/fimmu.2026.1861904 (PMC13534008; doi:10.3389/fimmu.2026.1861904)
Supplement: Supplementary file 1 [file DataSheet1.zip › Supplementary Materials/Supplementary Table S2.docx]

|  | **SLE_S** | **SLE_A** | **HC** |
| --- | --- | --- | --- |
| **Number of Subjects** | 82 | 48 | 90 |
| **Age (years, mean (SD))** | 40.4±12.3 | 37.2±13.4 | 39±10.5 |
| **Sex, Female** | 70 (85.4) | 40 (83.3) | 76 (84.4) |
| **Clinical Criteria:** |  |  | NA |
| **Rash** | 18 (22.0) | 14 (29.2) | NA |
| **Oral ulcers** | 0 (0) | 3 (6.3) | NA |
| **Nonscarring alopecia** | 2 (2.4) | 3 (6.3) | NA |
| **Synovitis involving two or more joints** | 8 (9.8) | 19 (39.6) | NA |
| **Serositis** | 3 (3.7) | 2 (4.2) | NA |
| **Renal disorder** | 14 (17.1) | 32 (66.7) | NA |
| **Neurologic disorder** | 0 (0) | 0 (0) | NA |
| **Leukopenia (< 4000/mm3 at least once)** | 7 (8.5) | 4 (8.3) | NA |
| **Thrombocytopenia (<100,000/mm3) at least once** | 2 (2.4) | 6 (12.5) | NA |
| **Immunological Criteria:** |  |  | NA |
| **Anti-dsDNA** | 26 (31.7) | 31 (64.6) | NA |
| **Anti-Sm** | 22 (26.8) | 15 (31.3) | NA |
| **Anti-β2GPI** | 5 (6.1) | 3 (6.3) | NA |
| **ACL-IgG** | 8 (9.8) | 5 (10.4) | NA |
| **ACL-IgM** | 1(1.2) | 2 (4.2) | NA |
| **Low complement (low C3)** | 22 (26.8) | 29 (60.4) | NA |
| **Low complement (low C4)** | 25 (30.5) | 22 (45.8) | NA |
| **Direct Coombs test in the absence of hemolytic anemia** | 9 (11.0) | 10 (20.8) | NA |

**Supplementary Table S2 Demographic and Clinical Characteristics of Healthy Controls (HCs) and Patients with Systemic Lupus Erythematosus (SLE)**
